# Effect of Online Clinic on Follow-Up Compliance and Survival Outcomes in Nasopharyngeal Carcinoma: Real-World Cohort Study from Endemic Area

**DOI:** 10.3390/healthcare12141452

**Published:** 2024-07-21

**Authors:** Siqi Chen, Chenyang Feng, Peng Sun, Jingrong Zhang, Hu Liang

**Affiliations:** 1 Lingnan College, Sun Yat-sen University, Guangzhou 510275, China; chensq36@mail2.sysu.edu.cn; 2 Sun Yat-sen University Cancer Center, State Key Laboratory of Oncology in South China, Collaborative Innovation Center for Cancer Medicine, Guangdong Key Laboratory of Nasopharyngeal Carcinoma Diagnosis and Therapy, Guangzhou 510060, China; fengcy1@sysucc.org.cn (C.F.); sunpeng1701@gmail.com (P.S.); 3 Information Technology Center, Sun Yat-sen University Cancer Center, Guangzhou 510060, China; 4 Department of Medical Oncology, Sun Yat-sen University Cancer Center, Guangzhou 510060, China; 5 Guangzhou Women and Children’s Medical Center, Guangzhou Medical University, Guangzhou 510623, China; 6 Department of Nasopharyngeal Carcinoma, Sun Yat-sen University Cancer Center, Guangzhou 510060, China

**Keywords:** online clinic, follow-up compliance, nasopharyngeal carcinoma, survival analysis, real-world cohort study

## Abstract

Nasopharyngeal carcinoma (NPC) requires regular follow-up to detect recurrence as early as possible. However, many patients are unable to regularly follow up due to the inconvenience of the conventional approach. Therefore, this study was designed to investigate the impact of the online clinic on follow-up compliance and prognosis in NPC patients. Patients who were first diagnosed with NPC between April 2019 and November 2019 were enrolled. Good follow-up compliance was defined as having at least one follow-up visit every 6 months within 2 years after treatment completion. Sensitivity analyses were performed using a propensity score matching model. A total of 539 (42%) patients used online follow-up while 731 (58%) used traditional follow-up. The median age of patients in the online cohort was lower than that in the traditional cohort (44 vs. 47, *p* < 0.001). Compared with the traditional cohort, the online cohort had significantly better follow-up compliance (57.3% vs. 17.1%, *p* < 0.001) and a higher 2-year PFS rate (98.1% vs. 94.4%, *p* = 0.003). Survival analysis showed that online follow-up was an independent factor for better survival prognosis (HR 0.39, 95%CI 0.20–0.74, *p* = 0.004). Sensitivity analysis further confirmed these results. Our study found that the online clinic increased follow-up compliance and improved prognosis in NPC patients.

## 1. Introduction

Nasopharyngeal carcinoma is a malignant tumor originating from the epithelial cells of the nasopharynx, with a high incidence in southern China, Southeast Asia, and North Africa [1,2]. The guidelines for tumor monitoring are based on expert opinions, recommending regular follow-up for survivors (i.e., undergoing physical and imaging examinations every 3–6 months on average) to promote the early detection of disease recurrence or metastasis [3,4,5,6]. The recurrence or metastasis of nasopharyngeal carcinoma was most common within 1 to 2 years after treatment completion in some studies [1,7]. Specifically, the cumulative metastatic rate at 2 years was 70.3% [8], and local and regional recurrences were most common between the 18th and 24th months [5]. Therefore, regular follow-up is particularly important for the early detection of disease relapse in nasopharyngeal carcinoma survivors. To promote regular follow-up in patients with nasopharyngeal carcinoma, guidelines and related studies provide specific recommendations for follow-up in recent years. These guidelines recommend a follow-up check every 3–6 months in the first 1–3 years after treatment [9,10]. Scholars have also proposed the most cost-effective follow-up strategy [3] and individual follow-up strategies based on their clinical stages [11] for patients with nasopharyngeal carcinoma.

Despite the follow-up strategies recommended in guidelines and related studies, few cancer survivors comply with follow-up recommendations after treatment completion. For example, in one study, fewer than 20% of patients had a medical visit for follow-up in the first 2 years, and 10% of patients never followed up in the Childhood Cancer Survivor Study [12,13]. Similarly, in real-world clinical practices, few patients with nasopharyngeal carcinoma comply with physicians’ regular follow-up strategies, indicating unsatisfactory compliance. Especially during the pandemic period, the number of follow-up visits dropped sharply. A study by the Lithuanian Cancer Center found that the pandemic outbreak reduced follow-up visits by 16% [14]. Therefore, new forms of follow-up are needed to improve patients’ follow-up compliance.

Digital medical technologies can enhance follow-up [15] by facilitating information storage [16] and can also improve patients’ compliance [17]. The internet hospital, which is an important part of digital medical technologies, is an innovative healthcare service model encouraged by the Chinese government [18]. The internet hospital enables patients to consult in a timely manner, which improves the accessibility, convenience, and efficiency of medical services. Therefore, digital medical technologies, especially the internet hospital, might be a good choice to promote cancer survivor for regular follow-up. In practice, the Sun Yat-sen University Cancer Center (shortened to “center”) established an internet hospital, called the online clinic, to provide patients with an online follow-up option so that follow-up patients of the center could use the online clinic for consultations and examination appointments. The impacts of digital medical technologies on different clinical trials are different. Some scholars have found that digital medical technologies facilitate disease detection [19,20]. However, other studies have indicated that digital medical technologies do not significantly reduce the average number of emergency department visits [21,22].

With regard to nasopharyngeal carcinoma, the online clinic provided convenience for patients, but its impact on follow-up compliance and survival outcomes remained unclear. Therefore, in this study, we aimed to assess the impact of online follow-up on patients’ follow-up compliance and progression-free survival (PFS). We expected that our study could provide suggestions for the follow-up of patients with nasopharyngeal carcinoma.

## 2. Materials and Methods

### 2.1. Study Cohort

This study was reviewed and approved by the Institutional Review Board and Ethics Committee of Sun Yat-sen University Cancer Center, Guangzhou, China (No. G2021-008-01). The electronic medical records and outpatient medical charts of patients diagnosed with nasopharyngeal carcinoma at the Sun Yat-sen University Cancer Center from April 2019 to November 2019 were reviewed. Written informed consent for the use of clinical data and collected samples for future studies (including retrospective studies) was obtained when the patients were admitted to receive treatment as a general standard procedure for patients treated in our center. All patient records were anonymous and de-identified before the analysis. This study was conducted in accordance with the Declaration of Helsinki.

**Figure 1:**
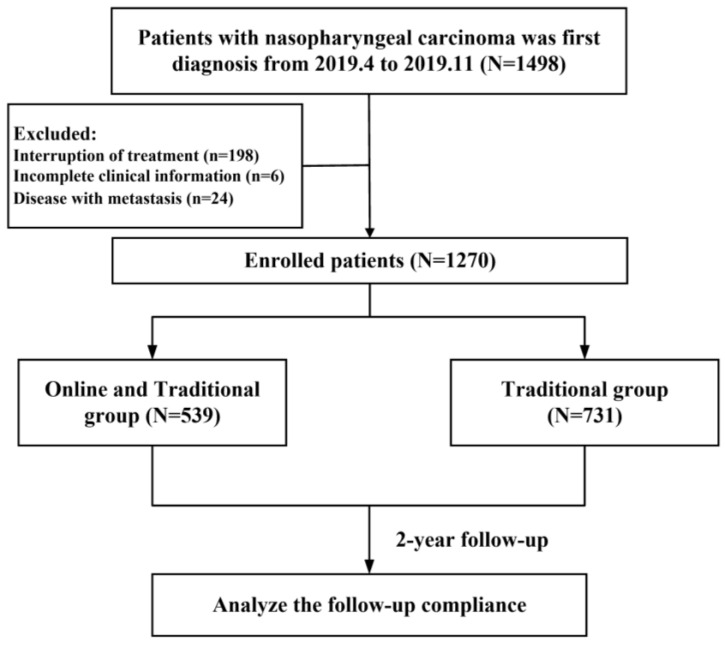
Flow chart.

The inclusion criteria for patients were as follows: histologically confirmed nasopharyngeal carcinoma, disease classified as stages II–IVA (AJCC 8 edition), receiving and completing radical treatment, no other life-threatening diseases, and completed follow-up data. The main exclusion criteria were as follows: interruption of treatment, incomplete clinical information, disease with metastasis. A total of 1270 patients were identified in the final statistical analysis, with 228 patients excluded. Detailed information about the study patients is shown in Figure 1.

### 2.2. Online Clinic Follow-Up Management System

In general, when a patient with nasopharyngeal carcinoma followed up, consultation, appointment, and examination were required. Notably, during the follow-up period, magnetic resonance imaging (MRI), computed tomography (CT), and other imaging examinations had to be performed to detect disease progression. Thus, all patients with nasopharyngeal carcinoma had to go to the center for examinations. In the traditional medical consultation model, patients had to make multiple trips to the center for follow-up, which caused financial pressure and mental burden, especially for patients living far from the center.

At the Sun Yat-sen University Cancer Center, a national key specialized cancer center, more than 85% of inpatients came from outside Guangzhou and over 85% of outpatients visited for follow-up in 2017. To enhance the center’s medical service capabilities and facilitate patients returning for follow-up, the center launched the first specialized online clinic in China in 2018, and this was fully promoted in 2020. The online clinic, which utilized internet cloud technology, provided the functions of consultation and appointment, so patients could use the online clinic for follow-up processes except examinations. With the emergence of the online clinic, the center’s patients could use the online clinic for consultations and appointments during follow-up. Then, physicians provided consultations and appointments based on patients’ electronic medical records and consultation information. Therefore, compared with the traditional consultation model, the number of visits to the center was reduced sharply when patients used the online clinic for follow-up. Once patients registered on the center’s online clinic, they could consult with a physician using any message types, including text, images, and voice.

The center’s patients could choose online or offline clinics for follow-up consultations and appointments, but they all had to go to the center for examinations. Therefore, we defined form of follow-up according to patients’ channels for consultations and appointments during the follow-up period. In our study, the follow-up period was 2 years after treatment completion. During the follow-up period, if a patient used a combination of the center’s online and offline clinics for consultations and appointments, and used the online clinic more than once, we defined the patient’s form of follow-up as online follow-up; otherwise, if a patient only used the center’s offline clinic for consultations and appointments, and did not use the online clinic, we defined the patient’s form of follow-up as traditional follow-up. Then, according to patients’ forms of follow-up during follow-up period, they were categorized into the online cohort and the traditional cohort. If a patient’s form of follow-up was online follow-up, the patient was categorized into the online cohort; otherwise, if a patient’s form of follow-up was traditional follow-up, the patient was categorized into the traditional cohort.

The pandemic caused significant inconvenience for the patients in our study, but the emergence of the center’s online clinic provided more opportunities for them to follow up. For example, they could use the online clinic for consultations and appointments. Based on the above analysis, all follow-up patients with nasopharyngeal carcinoma had to go to the center for examinations, which was challenging during the pandemic, especially for those far from the center. Guidelines recommended that patients followed up every 3–6 months after treatment completion. This meant the follow-up was not particularly urgent, so patients could make appointments for examinations in advance according to the actual situation. Patients who found it convenient to travel to the center for examinations could use the online clinic for consultations and examination appointments, then go to the center for examinations and consult the examination results through the online clinic. Patients who found it inconvenient to travel to the center for examinations could make appointments at hospitals near their homes for examination and then upload the examination results to the center’s online clinic for consultations. Therefore, the online clinic helped the center’s patients overcome some challenges for follow-up during the pandemic period.

### 2.3. Follow-Up

**Figure 2:**
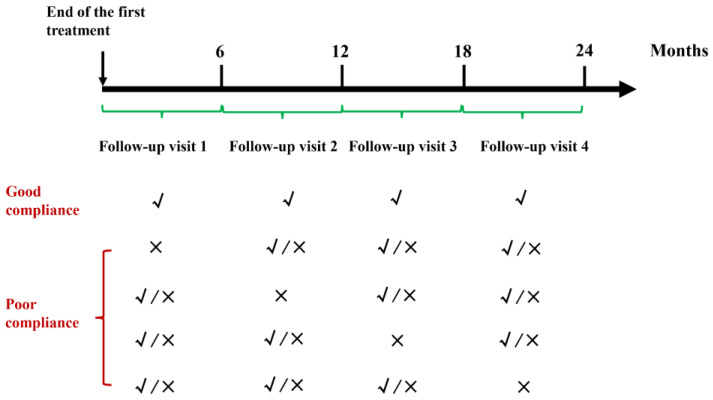
Description of good and poor follow-up compliance. √: A patient followed up at least once. ×: A patient did not follow up.

All patients were treated with radical concurrent chemoradiotherapy according to the principle of treatment for nasopharyngeal carcinoma at our cancer center. After the primary treatment, the patients were required to follow up every 3 or 6 months for the first 2 years. Considering the potential influence of the Coronavirus Disease 2019 pandemic, in the present study, the regular follow-up strategy was defined as visiting the center’s online or offline clinic at least once for follow-up every 6 months within 2 years after treatment completion. The follow-up compliance was divided into two kinds: good and poor. Good compliance was defined as completing the regular follow-up before death. Otherwise, patients who did not participate in the regular follow-up before death were considered to have poor compliance (Figure 2).

### 2.4. Outcome and Variable Definitions

The primary endpoints were the 2-year follow-up compliance and 2-year PFS between the two cohorts. PFS was defined as the interval from the date of diagnosis to the date of locoregional recurrence, distant metastasis, or death from any cause, whichever occurred first. Based on the electronic medical record, variables were extracted, including age, sex, history of concomitant disease, infectious diseases (i.e., hepatitis, tuberculosis), T category, N category, clinical stage, plasma EBV DNA levels, and distance from home to the center.

### 2.5. Statistical Analysis

Categorical variables were compared between the cohorts using the Chi-square test. Continuous variables were compared using the Mann–Whitney U test. Survival rates were estimated using the Kaplan–Meier method with the log-rank test. To assess the association between form of follow-up and follow-up compliance, the multivariate logistic regression analysis model was used with the forward likelihood ratio method. The Cox proportional hazards model was performed to assess the association between form of follow-up and 2-year PFS rate with the forward likelihood ratio method and assessed based on Schoenfeld residuals. In order to clarify the goodness of fit of the regression model, the Hosmer–Lemeshow test was performed, and a *p* value of more than 0.05 indicated the model’s good estimation. To enhance the credibility of results, the propensity score matching (PSM) model was used to adjust for confounders using calipers of a width equal to 0.2 standard deviations of the logit of the estimated propensity score.

All statistical tests were two-sided and a *p* value of less than 0.05 was considered statistically significant. All analyses were performed using SPSS (version 26.0, International Business Machines Corporation, Armonk, NY, USA) and STATA software (version 16.0, College Station, TX, USA, StataCorp LLC.).

## 3. Results

### 3.1. Patient Characteristics

A total of 1270 eligible patients were enrolled in the study (Figure 1), with a median age of 46 years (interquartile range [IQR], 37–54 years), including 922 (73%) males and 348 (27%) females. There were 101 (8%) patients with stages I–II and 1169 (92%) patients with stages III–IV. Specifically, the number of patients with T1–2, T3–4, N0–1, and N2–3 were 195 (16%), 1075 (84%), 533 (42%), and 737 (58%), respectively. With the cutoff of 2000 copies/mL of plasma EBV DNA levels, there were 940 (74%) patients with low EBV DNA levels and 330 (26%) patients with high EBV DNA levels. The most common comorbidity was hypertension (9%), followed by hepatitis (8%) and diabetes (4%). The median distance from home to the center was 350 km (IQR, 100 to 680 km).

Of the total 1270 patients, 539 patients (42%) belonged to the online cohort while 731 patients (58%) belonged to the traditional cohort (Table 1). Patients in the online cohort were younger than those in the traditional cohort (median age: 44 years [IQR, 36–52 years] vs. 47 years [IQR, 39–56 years]; *p* < 0.001). Compared with the online cohort, patients in the traditional cohort had higher comorbidity rates of hypertension (6% vs. 11%; *p* = 0.007), diabetes mellitus (2% vs. 5%; *p* = 0.007), and hepatitis infectious disease (7% vs. 10%; *p* = 0.05). Regarding the distance from home to the center, the online cohort had a significantly longer distance compared with the traditional cohort (median distance: 390 km [IQR, 140–680 km] vs. 350 km [IQR, 100–580 km]; *p* = 0.040). No differences were observed in sex, coronary heart disease, malignant tumors, tuberculosis, clinical stage, and EBV DNA levels between the two cohorts.

Considering the significant differences in characteristics (i.e., age [*p* < 0.001], hypertension [*p* = 0.007], diabetes [*p* = 0.007], hepatitis [*p* = 0.05], and distance from home to the center [*p* = 0.040]) affecting follow-up strategies and clinical outcomes, a propensity score matching model with observational factors was used to confirm and recalculate the prognostic effects of the two follow-up strategies. A total of 333 paired patients were identified in the matched analysis. The differences in all of the observed characteristics were eliminated in the matched analysis (Table 2).

### 3.2. Follow-Up Compliance

**Figure 3:**
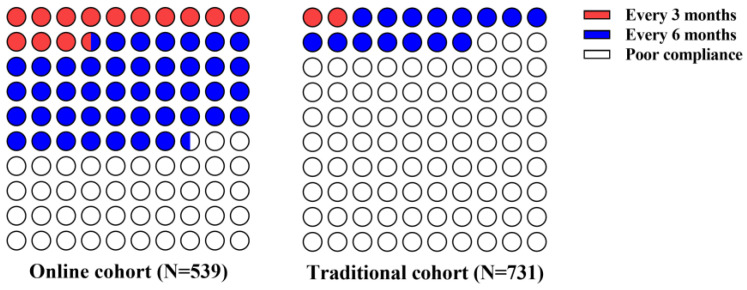
Follow-up compliance in online and traditional cohorts: the follow-up compliance in online (**left**) and traditional (**right**) cohorts. In the online cohort, 74 (13.7%) and 235 (43.6%) patients underwent regular follow-up every 3 months and every 6 months, respectively. In the traditional cohort, 14 (1.9%) and 111 (15.2%) patients underwent regular follow-up every 3 months and every 6 months, respectively.

**Figure 4:**
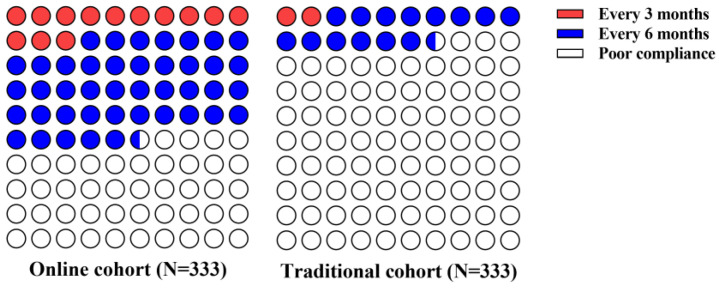
Follow-up compliance in online and traditional cohorts in PSM set: the follow-up compliance in online (**left**) and traditional (**right**) cohorts in the PSM set. In the online cohort, 43 (12.9%) and 141 (42.3%) patients underwent regular follow-up every 3 months and every 6 months, respectively. In the traditional cohort, 6 (1.8%) and 49 (14.7%) patients underwent regular follow-up every 3 months and every 6 months, respectively.

Of the total 1270 patients, there were 731 (58%) patients in the traditional cohort and 539 (42%) patients in the online cohort. Compared with the traditional cohort, the online cohort had more patients with good follow-up compliance (309/539 [57.3%] vs. 125/731 [17.1%]; *p* < 0.001). Specifically, the proportion of patients with at least one follow-up visit every 3 months and the proportion of patients with at least one follow-up visit every 6 months were both significantly higher in the online cohort than in the traditional cohort (74 [13.7%] vs. 14 [1.9%] and 235 [43.6%] vs. 111 [15.2%], respectively; both *p* < 0.001; Figure 3). Similar results were observed in the matched analysis (Figure 4).

Then, logistic regression analysis was performed to assess the association between form of follow-up and follow-up compliance. In the univariate analyses, online follow-up (OR: 6.45, 95%CI: 4.99–8.34, *p* < 0.001) and being under 45 years old (OR: 0.67, 95%CI: 0.53–0.85, *p* = 0.001) were associated with good follow-up compliance. In the multivariate analysis, online follow-up (OR: 6.32, 95%CI: 4.88–8.18, *p* < 0.001) was independently associated with good follow-up compliance (Table 3). Similar results were observed in the matched analysis (Table 4).

### 3.3. Survival Analysis

Cox proportional hazards regression analysis was performed to assess the association between the form of follow-up and 2-year PFS rate. In the univariate analyses, traditional follow-up (HR: 0.39, 95%CI: 0.20–0.74, *p* = 0.004), being over 45 years old (HR: 1.78, 95%CI: 1.00–3.16, *p* = 0.05), and a higher clinical stage (HR: 2.19, 95%CI: 1.33–3.60, *p* = 0.002) were associated with a lower 2-year PFS rate. Then, statistically significant variables (*p* ≤ 0.05) in the univariate analysis (i.e., form of follow-up, age, and clinical stage) were included in the multivariate analysis. Finally, we found that traditional follow-up (HR: 0.40, 95%CI: 0.21–0.76, *p* = 0.005) and a higher clinical stage (HR: 2.25, 95%CI: 1.37–3.70, *p* = 0.001) were associated with a lower 2-year PFS rate in the multivariate analysis (Table 5). Similar results were observed in the matched analysis (Table 6).

**Figure 5:**
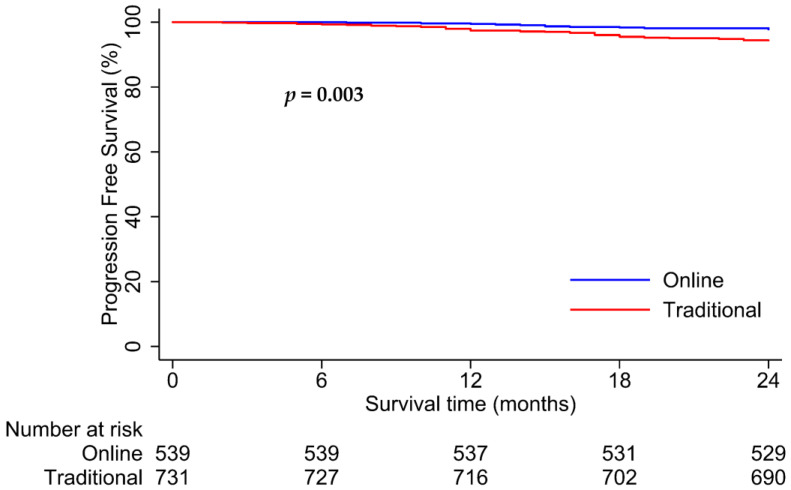
Kaplan–Meier curves for progression-free survival in online and traditional cohorts. The online cohort (blue line) had significantly higher progression-free survival than the traditional cohort (red line), with 2-year PFS rates of 98.1% and 94.4%, respectively.

**Figure 6:**
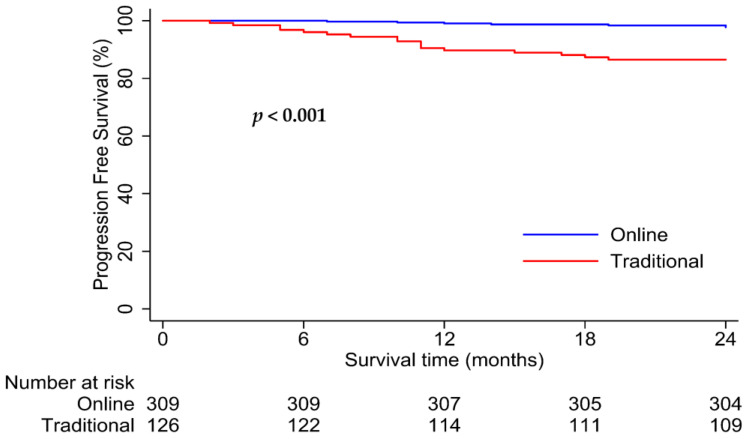
Kaplan–Meier curves for progression-free survival in online and traditional cohorts with good compliance. The online cohort (blue line) had significantly higher progression-free survival than the traditional cohort (red line), with 2-year PFS rates of 98.4% and 86.5%, respectively.

**Figure 7:**
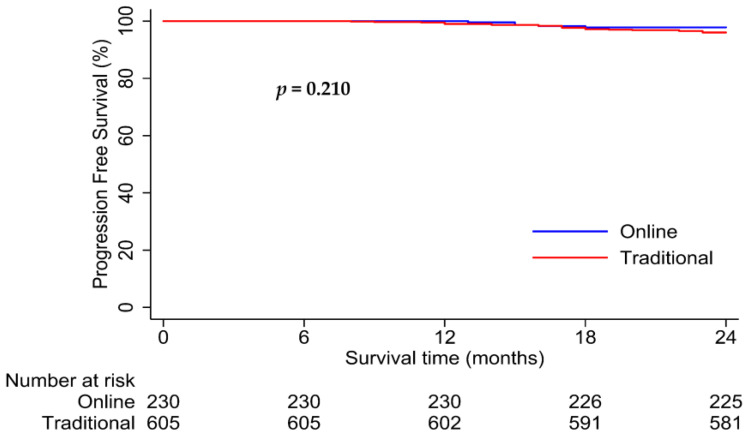
Kaplan–Meier curves for progression-free survival in online and traditional cohorts with poor compliance. No significant difference in survival was observed between the two cohorts.

**Figure 8:**
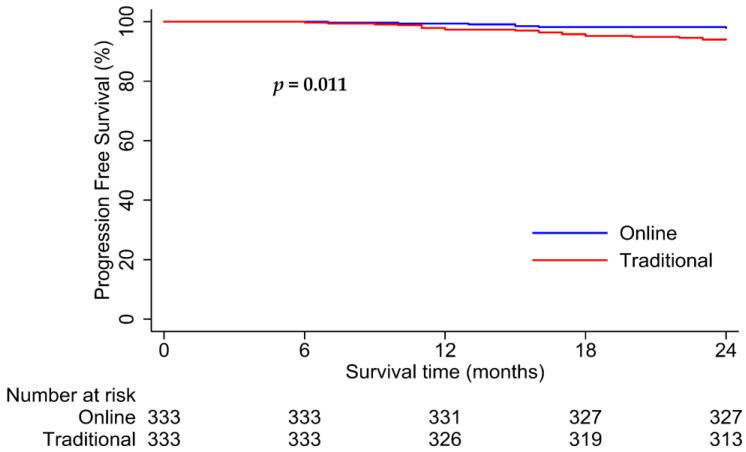
Kaplan–Meier curves for progression-free survival in online and traditional cohorts in PSM set. The online cohort (blue line) had significantly higher progression-free survival than the traditional cohort (red line), with 2-year PFS rates of 98.2% and 94%, respectively.

Patients in the online and traditional cohorts had 2-year PFS rates of 98.1% versus 94.4%, respectively (*p* = 0.003; Figure 5). Interestingly, for patients with good follow-up compliance, the online cohort had significantly better PFS than the traditional cohort (2-year PFS rates: 98.4% vs. 86.5%, *p* < 0.001; Figure 6). However, no significant difference in survival was observed between the two cohorts with poor follow-up compliance (2-year PFS rates: 97.8% vs. 96.0%, *p* = 0.21; Figure 7). Similar results were observed in the matched analysis. Specifically, patients in the online and traditional cohorts had 2-year PFS rates of 98.2% versus 94%, respectively (*p* = 0.011; Figure 8).

## 4. Discussion

To the best of our knowledge, this study was the first to show that the online clinic significantly improved follow-up compliance in patients with nasopharyngeal carcinoma who received the radical treatment and improved the survival of patients with online follow-up compared with traditional follow-up.

Previous studies showed that the regular follow-up of cancer patients was beneficial for improving prognosis [5,23], so follow-up strategies for cancer patients received much attention. For example, many studies provided personalized and cost-effective follow-up strategies for patients with nasopharyngeal carcinoma [5,11,24], esophageal cancer [25], and hepatocellular carcinoma [26]. Moreover, the online clinic improved follow-up compliance and might help improve the 2-year PFS rate. In this study, we initially found that online follow-up was associated with a higher 2-year PFS rate in patients with nasopharyngeal carcinoma. We would further investigate whether follow-up compliance mediated the relationship between the form of follow-up and 2-year PFS rate in patients with nasopharyngeal carcinoma in subsequent studies.

Meanwhile, our study period spanned the pandemic and was undoubtedly affected by it. During the pandemic period, it was inconvenient for patients to follow up, and the follow-up visits dropped significantly [14]. Telemedicine eased the challenges of accessing medical treatment for patients with cancer during the pandemic, such as by ensuring the sustainability of medical service [27], improving the health outcomes of patients by detecting complications earlier, and reducing costs [27,28]. Obviously, the pandemic was a social factor promoting the implementation of telemedicine and the utilization rate of telemedicine increased significantly [29]. In practice, online follow-up provided more opportunities for patients with nasopharyngeal carcinoma to follow up, which facilitated patients to follow up as regularly as possible during the pandemic period. The center’s online clinic provided patients with the functions of consultation and examination appointment. Guidelines recommended that patients followed up every 3–6 months, which meant that follow-up was not particularly urgent, so patients could make appointments in advance according to the actual situation. During the pandemic, if patients found it convenient to travel to the center for examinations, they could use the online clinic for consultations and examination appointments, then go to the center for examinations and consult the examination results through the online clinic. If patients found it inconvenient to travel to the center for examinations, they could be examined at local hospitals near their homes and then upload the examination results to the center’s online clinic for consultations.

The online clinic enabled patients to realize remote consultation [30], and it was used for follow-up management, which improved the patients’ quality of life [31], improved compliance [32], helped the patients cope better with the disease [33], and solved most issues effectively [34]. The analysis found that patients in the online cohort, who used a combination of online and offline clinics for follow-up, were younger, and online follow-up was beneficial to patients with nasopharyngeal carcinoma. Therefore, the center should take relevant measures to increase the interest of elderly patients and patients with limited mobility in online follow-up. Firstly, physicians should emphasize the importance of regular follow-up with patients and encourage them to use a combination of online and offline clinics for follow-up [35]. Secondly, the center could design a simple and convenient interactive interface for the online clinic and proactively guide patients on its use. Finally, the center could appropriately provide patients with free online clinic opportunities to try and experience how it works. In addition, the center could also take relevant surveys to understand patient satisfaction and demand. Indeed, online follow-up facilitated patients’ follow-up [31,33,34] and reduced the disease progression rate via the earlier detection of signs of tumor recurrence and metastasis [33], which were supported by our results. In the future, we would collect more data on patients’ socio-economic backgrounds and further explore their impact on follow-up compliance and prognosis.

In the future, combining the online clinic with advanced technologies, such as artificial intelligence (AI) and machine learning (ML), is a promising development direction. Firstly, the online clinic could be combined with AI to reduce differences in the interpretation of other hospitals’ examination reports. Imaging examinations are necessary to detect disease progression [36], but the detection accuracy and interpretation of examination reports are affected by the reading experience of the radiologist in question [37]. Using models based on AI could improve the detection accuracy and reduce differences in the interpretation of other hospitals’ examination reports [38]. Secondly, the online clinic could combine AI and ML to monitor patients’ disease progression and provide personalized treatment. Patients with cancers in this study needed to monitor their symptoms to detect disease recurrence and metastasis as early as possible, but symptoms were often undetected and untreated [39,40,41]. The online clinic could further expand its functions—for example, by collecting regular patient-reported outcome (PRO) surveys [20] to efficiently monitor patients’ symptoms [42] at a low cost [43] and improve the health-related quality of life [20,42,44,45], as well as by collecting patients’ daily data, such as on their heart rates, through wearable devices [19,46,47,48,49]. Further, the online clinic could use ML models to predict patients’ survival outcomes based on the above data [50,51] and then provide personalized care [27,52] and patient-centered treatment, ultimately improving the quality of life of cancer survivors [50,53].

In addition, our study had some limitations. Firstly, this study was retrospective and may have been affected by data completeness and selection bias. Secondly, this study was a single-center retrospective study with a small sample size and limited generalizability of results, so subsequent multi-center studies are needed to obtain generalizable results. Thirdly, our study period spanned the pandemic, which might have had some impact on the overall follow-up, leading to some population selection bias. However, it was also due to the existence of the pandemic that the value of the online clinic was demonstrated. Further, this study’s results show that the online clinic helps reduce the impact of large-scale emergencies, such as pandemics, on regular follow-up. Fourthly, due to the absence of prior studies on follow-up compliance in nasopharyngeal carcinoma, we set follow-up compliance to 3–6 months based on the clinical guidelines, which was not a traditional setting method. Fifthly, this study was based on conclusions drawn from a nasopharyngeal carcinoma cohort, and the impact of the online clinic on the follow-up of other diseases remains to be verified. Sixth, we lacked some data on patients’ socio-economic backgrounds. In the future, we will collect data on patients’ socio-economic backgrounds and explore their impact.

In conclusion, online follow-up improves follow-up compliance in patients with nasopharyngeal carcinoma and improves progression-free survival through early detection. This study suggests that physicians can encourage patients with nasopharyngeal carcinoma to use a combination of offline and online clinics for follow-up consultations.

## Figures and Tables

**Table 1:** Baseline clinical characteristics.

Characteristics	Total (N = 1270)	Online Cohort (N = 539)	Traditional Cohort (N = 731)	*p* Value
Age				
Median age (years)	46 (37 to 54)	44 (36 to 52)	47 (39 to 56)	<0.001
Sex				0.638
Male	922 (73%)	395 (73%)	527 (72%)	
Female	348 (27%)	144 (27%)	204 (28%)	
Pre-existing conditions				
Hypertension	112 (9%)	34 (6%)	78 (11%)	0.007
Coronary heart disease	8 (<1%)	4 (<1%)	4 (<1%)	0.664
Diabetes	47 (4%)	11 (2%)	36 (5%)	0.007
Malignant tumors	7 (<1%)	2 (<1%)	5 (<1%)	0.457
Infectious diseases				
Hepatitis	107 (8%)	36 (7%)	71 (10%)	0.054
Tuberculosis	28 (2%)	13 (2%)	15 (2%)	0.666
T category				0.859
T1	59 (5%)	23 (4%)	36 (5%)	
T2	136 (11%)	54 (10%)	82 (11%)	
T3	752 (59%)	324 (60%)	428 (59%)	
T4	323 (25%)	138 (26%)	185 (25%)	
N category				0.217
N0	114 (9%)	53 (10%)	61 (8%)	
N1	419 (33%)	166 (31%)	253 (35%)	
N2	494 (39%)	206 (38%)	288 (39%)	
N3	243 (19%)	114 (21%)	129 (18%)	
Clinical stage				0.694
I	25 (2%)	9 (2%)	16 (2%)	
II	76 (6%)	29 (5%)	47 (7%)	
III	635 (50%)	267 (50%)	368 (50%)	
IV	534 (42%)	234 (43%)	300 (41%)	
EBV DNA				0.121
≤2000 copies/mL	940 (74%)	411 (76%)	529 (72%)	
>2000 copies/mL	330 (26%)	128 (24%)	202 (28%)	
Distance from home to the center (km)	350 (100 to 680)	390 (140 to 680)	350 (100 to 580)	0.040

Data are shown by median (IQR) or n (%) values. Abbreviations: EBV, Epstein–Barr virus.

**Table 2:** Baseline clinical characteristics in PSM set.

Characteristics	Total (N = 666)	Online Cohort (N = 333)	Traditional Cohort (N = 333)	*p* Value
Age				
Median age (years)	48 (39 to 55)	46 (39 to 55)	46 (37 to 55)	0.541
Sex				0.678
Male	453 (68%)	229 (69%)	224 (67%)	
Female	213 (32%)	104 (31%)	109 (33%)	
Pre-existing conditions				
Hypertension	74 (11%)	32 (10%)	42 (13%)	0.218
Coronary heart disease	6 (1%)	3 (1%)	3 (1%)	>0.999
Diabetes	23 (3%)	11 (3%)	12 (4%)	0.832
Malignant tumors	6 (1%)	2 (1%)	4 (1%)	0.412
Infectious diseases				
Hepatitis	70 (11%)	35 (11%)	35 (11%)	>0.999
Tuberculosis	18 (3%)	10 (3%)	8 (2%)	0.633
T category				0.460
T1	40 (6%)	18 (5%)	22 (7%)	
T2	78 (12%)	34 (10%)	44 (13%)	
T3	383 (57%)	200 (60%)	183 (55%)	
T4	165 (25%)	81 (25%)	84 (25%)	
N category				0.217
N0	57 (9%)	27 (8%)	30 (9%)	
N1	242 (36%)	115 (35%)	127 (38%)	
N2	239 (36%)	118 (35%)	121 (36%)	
N3	128 (19%)	73 (22%)	55 (17%)	
Clinical stage				0.336
I	17 (2%)	8 (2%)	9 (3%)	
II	59 (9%)	23 (7%)	36 (11%)	
III	311 (47%)	157 (47%)	154 (46%)	
IV	279 (42%)	145 (44%)	134 (40%)	
EBV DNA				0.561
≤2000 copies/mL	499 (75%)	246 (74%)	253 (76%)	
>2000 copies/mL	167 (25%)	87 (26%)	80 (24%)	
Distance from home to center (km)	350 (100 to 680)	380 (100 to 680)	350 (100 to 680)	0.602

Data are shown by median (IQR) or n (%) values. Abbreviations: EBV, Epstein–Barr virus. Factors, such as age, sex, hypertension, coronary heart disease, diabetes, malignant tumors, hepatitis, tuberculosis, T category, N category, clinical stage, EBV DNA, and distance from home to center, were included in PSM set.

**Table 3:** Multivariate analysis for follow-up compliance with logistic model.

Characteristics	Univariate		Multivariate	
	OR (95%CI)	*p* Value	OR (95%CI)	*p* Value
Form of follow-up (Online, Traditional)	6.45 (4.99–8.34)	<0.001	6.32 (4.88–8.18)	<0.001
Age (≥45, <45)	0.67 (0.53–0.85)	0.001	0.78 (0.60–1.00)	0.052
Sex (Male, Female)	1.04 (0.80–1.35)	0.771		
Clinical stage	1.17 (0.98–1.40)	0.077		
Pre-existing condition (Yes, No)	0.93 (0.73–1.19)	0.586		

Variables with *p* ≤ 0.05 in the univariate regression analysis with logistic model were included in the multivariate regression analysis. Abbreviations: OR, odd ratio; CI, confidence interval.

**Table 4:** Multivariate analysis for follow-up compliance with logistic model in PSM set.

Characteristics	Univariate		Multivariate	
	OR (95%CI)	*p* Value	OR (95%CI)	*p* Value
Form of follow-up (Online, Traditional)	6.24 (4.35–8.96)	<0.001	6.81 (4.69–9.90)	<0.001
Age (≥45, <45)	0.94 (0.68–1.30)	0.703	0.66 (0.45–0.96)	0.029
Sex (Male, Female)	0.87 (0.62–1.22)	0.429		
Clinical stage	1.07 (0.86–1.33)	0.561		
Pre-existing condition (Yes, No)	0.98 (0.71–1.35)	0.903		

Age and form of follow-up were included in the multivariate regression analysis. Abbreviations: OR, odd ratio; CI, confidence interval.

**Table 5:** Multivariate analysis for 2-year PFS rate with Cox model.

Characteristics	Univariate		Multivariate	
	HR (95%CI)	*p* Value	HR (95%CI)	*p* Value
Compliance (Good, Poor)	1.63 (0.95–2.80)	0.077		
Form of follow-up (Online, Traditional)	0.39 (0.20–0.74)	0.004	0.40 (0.21–0.76)	0.005
Age (≥45, <45)	1.78 (1.00–3.16)	0.051	1.67 (0.93–2.97)	0.084
Sex (Male, Female)	1.88 (0.92–3.84)	0.086		
Clinical stage	2.19 (1.33–3.60)	0.002	2.25 (1.37–3.70)	0.001
Pre-existing condition (Yes, No)	1.34 (0.78–2.32)	0.289		

Variables with *p* ≤ 0.05 in the univariate regression analysis with Cox model were included in the multivariate regression analysis with Cox model. Abbreviations: HR, hazard ratio; CI, confidence interval.

**Table 6:** Multivariate analysis for 2-year PFS rate with Cox model in PSM set.

Characteristics	Univariate		Multivariate	
	HR (95%CI)	*p* Value	HR (95%CI)	*p* Value
Compliance (Good, Poor)	1.25 (0.58–2.69)	0.571		
Form of follow-up (Online, Traditional)	0.34 (0.15–0.81)	0.015	0.33 (0.14–0.78)	0.011
Age (≥45, <45)	1.21 (0.54–2.68)	0.647		
Sex (Male, Female)	1.67 (0.67–4.13)	0.270		
Clinical stage	1.92 (1.01–3.63)	0.045	1.99 (1.05–3.75)	0.034
Pre-existing condition (Yes, No)	1.05 (0.49–2.27)	0.896		

Variables with *p* ≤ 0.05 in the univariate regression analysis with Cox model were included in the multivariate regression analysis with Cox model. Abbreviations: HR, hazard ratio; CI, confidence interval.

## Data Availability

Data cannot be shared publicly because of the privacy implication, but data are available upon request and according to the national policy of data sharing, requiring authorization by the ethics committee.
